# Cache-efficient and vectorized parallel dynamic programming for RNA folding

**DOI:** 10.1371/journal.pone.0349146

**Published:** 2026-05-20

**Authors:** Mateusz Gruzewski, Marek Palkowski

**Affiliations:** West Pomeranian University of Technology in Szczecin, Szczecin, Poland; HSE University, RUSSIAN FEDERATION

## Abstract

In this article, we present an efficient and concise OpenMP implementation of the Nussinov RNA folding algorithm, a well-known representative of non-serial polyadic dynamic programming (NPDP). Our goal is to develop an optimized implementation that can serve as a template for related dynamic programming applications. The proposed code is derived from a detailed analysis of manual implementations, emphasizing the separation of problematic and non-problematic instances and structuring computations in a way analogous to matrix multiplication. This design enables the semi-automatic extraction of data locality using tools based on Presburger arithmetic—techniques widely employed in classical loop transformations and advanced source-to-source compilers grounded in the polyhedral model. In the experimental evaluation, we assess the performance of our implementation on modern massively parallel AMD and Intel processors with 64, 128, and 192 threads. Our approach leverages cache-aware tiling, parallelism, and explicit vectorization to maximize computational efficiency, achieving performance that surpasses both automatically generated compiler-based solutions and manually tuned implementations on the evaluated platforms. Specifically, our implementation achieves execution times up to two orders of magnitude faster than polyhedral code, while also outperforming unvectorized manual approaches—being at least 30 × faster than array transposition–based methods and at least 5 × faster than the tiled sparsified Four Russians variant. Additionally, our results indicate that CPU implementations do not exhibit significantly worse performance compared to their corresponding GPU counterparts. These results demonstrate the importance of leveraging Advanced Vector Extensions (AVX) to fully exploit the capabilities of modern multi-core processors, particularly those in the AMD Epyc family.

## Introduction

Dynamic programming (DP) is a method used in algorithm design to solve problems by breaking them down into smaller overlapping subproblems, solving each subproblem once, and storing the results to avoid redundant computations. It is especially useful for optimization problems where the solution to a larger problem depends on the solutions to smaller ones, a property known as optimal substructure [1]. Classic examples include the knapsack problem, matrix chain multiplication, and longest common subsequence.

Non-serial polyadic dynamic programming (NPDP) is a more general and abstract class of dynamic programming. Unlike standard dynamic programming, which typically combines two subproblems in a fixed, serial order, non-serial polyadic DP allows the combination of multiple subproblems in flexible and non-sequential ways. The term “polyadic” refers to combining more than two subproblems at once, while “non-serial” indicates that there is no strict left-to-right or sequential dependency between subproblems [2,3]. This framework includes a wide range of complex problems, such as optimal triangulation of polygons, matrix chain multiplication, optimal binary search trees, and often relies on specialized optimization techniques like Knuth’s optimization to achieve better performance [4–6].

The algorithms mentioned above share the same structural pattern as many bioinformatics approaches, particularly those based on the Nussinov RNA folding algorithm [7–10]. In these methods, for a given pair of indices *i*, *j*, the solution involves examining possible partitions within that interval by summing the results of subproblems across the row and column of the dynamic programming table, continually scanning all valid splits. In the case of Nussinov, this same dependency grid appears in the prediction of the secondary structure of the RNA, where the algorithm computes the best possible base pairings by seeking solutions to smaller subproblems. This dependency structure is also preserved in more advanced Nussinov-style algorithms in bioinformatics, such as the McCaskill algorithm to calculate the probability of base pairs [7] and MEA (Maximum Expected Accuracy) [11], and the Zuker algorithm for minimizing free energy [12], which all depend on traversing and combining DP entries in similar polyadic non-serial patterns (see S1 Table).

A typical parallelization strategy for such problems involves the execution of independent tasks along the diagonals of the dynamic programming table. This ordering respects the data dependencies that arise from reading values across rows and columns. Such diagonal processing enables concurrency without violating correctness. In compiler theory, this transformation is known as *loop skewing*, a fundamental loop transformation technique that restructures iteration space to expose parallelism. The DP table constructed in these algorithms is usually triangular in shape, reflecting the fact that solutions are only defined for entries where *i* < *j*, which is common in problems that involve pairing, chaining, or segmentation over sequences. One of the early works by Rizk and Lavinière [13] proposed an algorithm for processing along diagonals for both CPUs and GPUs. Achieving high-performance NPDP code that effectively exploits data locality and vectorization remains a challenging task [14–16].

Our goal is to develop a high-performance implementation of the Nussinov RNA folding algorithm, applicable to similar dynamic programming problems. The proposed solution leverages multicore CPUs and vectorization to achieve performance exceeding both automatic compiler optimizations and some manual implementations.

The paper is organized as follows. The Introduction presents the Nussinov NPDP algorithm with attention to data locality, briefly reviews polyhedral tools and manual methods, and outlines existing limitations and motivations. The next section introduces our approach based on dependency reduction, classification of problem instances, and automated code generation. The experimental section evaluates runtime efficiency on three multicore systems and compares results with related works. Finally, the conclusion summarizes findings and discusses future research directions.

### Nussinov’s algorithm

Nussinov proposed a dynamic programming algorithm for RNA folding in 1978 [8], which maximizes the number of non-crossing matchings between complementary bases of an RNA sequence of length *N*. Let $X=x_{1},x_{2},\ldots ,x_{N}$ be an RNA sequence, where $x_{i}\in \{G(\text{guanine}),A(\text{adenine}),U(\text{uracil}),C(\text{cytosine})\}$ is a nucleotide. Nussinov’s dynamic programming recurrence for an *N* × *N* matrix *S* is given below.

1 for (int i = N – 1; i >= 0; i--) {

2  for (int j = i + 1; j < N; j++) {

3   for (int k = i; k < j; k++) {

4     S[i][j] = max(S[i][k] + S[k + 1][j], S[i][j]); // S1

5   }

6   S[i][j] = max(S[i][j], S[i + 1][j-1] + paired(seqq[i], seqq[j])); // S2

7  }

8 }


$$\begin{array}{c} S(i,j)=\max_{\begin{array}{c} 1\leq i<j\leq N \end{array}}\left\{ \begin{array}{c} S(i+1,j-1)+\sigma (i,j),\\ \max_{i<k<j}\left(S(i,k)+S(k+1,j)\right) \end{array}\right. \end{array}$$ (1)

Here, *S*(*i*, *j*) defines the maximum number of base-pair matches of $x_{i},\ldots ,x_{j}$ over the region 1 ≤ *i* ≤ *N*, and σ(i,j) is a function that returns 1 if (*x*_*i*_, *x*_*j*_) is an AU, GC, or GU pair, and 0 otherwise.

Listing 1 represents the triply nested affine loops with two statements accessing the two-dimensional array *S* implementing Nussinov’s algorithm.

**Listing 1.** The Nussinov algorithm code.

The iteration space (*i*, *j*) of the program loop forms the upper right triangle of Nussinov’s dynamic programming array. The loop indices are defined over the domain *(i, j, k):*


$$\begin{array}{c} \left\{[i,j,k]\;|\;0\leq i<j<N,\;i\leq k<j\right\} \end{array}$$

### Locality improvement

The Nussinov algorithm and the family of related algorithms have a time complexity of *O*(*n*^3^) and a space complexity of *O*(*n*^2^). This means that during computation, the same cells in the cost (or pairing) table are visited multiple times, both row-wise and column-wise. To make better use of the cache, it is more efficient to use data blocking (also known as tiling), since the same rows and columns are repeatedly accessed [17]. By computing an entire block of target table entries at once rather than a single cell at a time, memory locality and cache utilization are improved, regardless of whether the computation is performed on a CPU or a GPU.

### Manual loop tiling

Rizk et al. [13] employed a piecewise-linear scheduling approach, as used in our work, to generate efficient GPU implementations for RNA folding. In [17], they extended this work to accommodate GPU blocks scanned diagonally. However, they do not provide a method for deriving this schedule from the program’s dependence structure [18].

Mullapudi and Bondhugula [2] have investigated automatic techniques for tiling codes that extend beyond the scope of standard methods. Their approach leverages dynamic tile scheduling instead of generating a static schedule, and it is applicable to Nussinov’s algorithm. At present, we lack a precise characterization of the specific domains where each technique proves most effective [18].

Li, Ranka, and Sahni [19] provided an efficient GPU algorithm that employs a matrix multiplication approach using a CUDA strategy for Nussinov’s RNA folding. In contrast, their CPU implementation is limited to a transpose-based technique that utilizes a lower-triangular array or row scanning. However, as demonstrated in‌‌ [16], this strategy proves inefficient for larger RNA strands. Furthermore, we also did not have access to the original GPU implementation of this achievement.

Zhao and Sahni developed three cache-efficient algorithms without increasing the memory requirement, *ByRow*, *ByRowSegment*, and *ByBox* for Nussinov’s RNA folding [20]. They showed that presented techniques based on a simple LRU cache model give better run time and energy performance than Li’s approach. Unfortunately, the code was limited to serial.

An efficient implementation has been demonstrated using the Four Russians (4R) strategy, which eliminates redundant computations by exploiting the fact that Nussinov’s array grows only until its last element [21,22]. 4R allows for a reduction in computational complexity by using a memoization strategy. However, as shown in [16], this work focuses only on the Nussinov algorithm, and no code for a parallel CPU version has been released. Moreover, if the NPDP algorithm produces an array in which elements increase and decrease, as in probabilistic RNA folding [7] - Venkatachalam’s precomputation cannot be applied directly. In 2023, Tchendji et al. [23] refined the technique by integrating a loop tiling strategy [9] with the sparsified Four Russians approach [21], implementing it using a portable OpenCL solution for both CPUs and GPUs for Nussinov’s code. Nonetheless, subsequent studies [16] indicate that this solution does not match the efficiency of CUDA-based implementations such as those proposed by Venkatachalam [22] and Gruzewski [16].

Wonnacott [18] presented a strategy for partitioning the domain of Nussinov’s instructions into problematic and non-problematic tiles. The non-problematic tiles can be executed arbitrarily, with the overall code execution synchronized to the completion of the problematic blocks. The paper demonstrates how to derive such a schedule through dependency analysis. Regrettably, the authors did not provide a CPU-based parallel implementation.

### Source-to-source polyhedral compilers

Simultaneously with the development of manually implemented blocked code for NPDP algorithms, automatic source-to-source polyhedral compilers emerged. These compilers primarily leverage approaches based on affine transformations, index set splitting, and the extraction of synchronization-free slices via Presburger arithmetic methods developed for code parallelization by Pugh’s team, in conjunction with the Omega Calculator library [24].

Standard operations on relations and sets are used, such as intersection (∩), union (∪), difference (-), composition (∘), domain (dom *R*), range (ran *R*), inverse (*R*^−1^), relation application (*S*$\backslash {(}^{\prime }$ = *R*(*S*): *e*$\backslash {(}^{\prime }\in {\text{S}}^{\prime }$ iff exists *e* s.t. *e* → *e*$\backslash {(}^{\prime }\in$*R*,*e* ∈ *S*). In detail, the description of these operations is presented in papers [25–27].

Based on this mathematical apparatus, the polyhedral model was developed. This framework is used to represent and optimize loop nests and array accesses in programs. In this model, iterations of loops are represented as points in a multidimensional integer space, and their execution domains are defined by polyhedra—geometric constructs characterized by linear inequalities. Data accesses and dependencies are expressed as affine functions of the loop indices, allowing the computation to be modeled in a precise, algebraic form.

This formulation enables a broad spectrum of optimizations, including loop tiling, fusion, parallelization, and reordering, by conceptualizing these operations as transformations on the underlying polyhedra. Because these transformations must adhere to data dependency constraints, the polyhedral model facilitates the application of integer linear programming and Presburger arithmetic techniques to ensure that any transformed schedule remains both correct and optimal. Ultimately, many modern automatic source-to-source compilers rely on the polyhedral model, leveraging its structured and rigorous representation of a program’s computational domain to generate highly efficient and parallelized code.

The Omega project was rewritten in the ISL framework by Sven Verdoolaege, incorporating a PET analyzer and a code generator, as well as standalone transformation engines [27]. Subsequent versions of the Pluto [28], Traco [9], and Dapt [29] compilers relied on this library, differing mainly in the specific loop transformation algorithms they employed.

The Pluto optimizer, when using the Affine Transformation Framework (ATF), encountered difficulties in tiling all loops in the Nussinov algorithm; in particular, it was unable to tile the innermost loop in s1, which is the most performance-critical. It also failed to parallelize the tiled code of the McCaskill algorithm for partition functions computation [10] because dependencies between statements in the strongly connected component (SCC – a maximal mutually reachable statements in dependence graph) prevent permutability in the parallel direction. In simpler terms, within an SCC, the statements are mutually dependent, forming a cycle of dependencies.

It was only with the Traco compiler, which applied tile corrections via transitive closure of the dependence graphs, that the tiling of all Nussinov loops was achieved. Later, the approach to NPDP algorithms was further refined through space-time tiling, thereby avoiding the costly tile correction, and this method was ultimately implemented in the Dapt tool.

### Limitations of existing Nussinov code optimization methods and objectives of this work

Despite numerous studies on both manual and automatic optimization of the Nussinov algorithm, several limitations remain. Existing methods lack a universal approach that can be easily applied to similar algorithms. They also do not provide efficient ways to exploit vectorization on modern CPU architectures, nor do they offer high-performance solutions that work across both GPU and CPU platforms. While efficient 4R [22,30] methods exist, their implementations are complex and largely restricted to the Nussinov algorithm on GPUs. Wonnacott’s [18] and Zhao’s [20] approaches produce only serial code, while Li’s implementation for CPU *Transpose* can be easily adapted to other problems but is not very effective. Polyhedral compilers [9,28,29] and parallel tiled sparsified 4R OpenCL implementations [14] have debatable performance. Hence, this motivated us to propose a solution meeting the following criteria:

- develop a simple and portable codebase that runs efficiently across different multi-core architectures,
- leverage both parallelism and vectorization to improve performance,
- ensure the approach is easily adaptable to similar NPDP computations, providing a flexible solution with short parallel code.

Finally, we will present an OpenMP [31] implementation that can be easily translated to other multi-threading libraries, demonstrating the portability and adaptability of our approach.

### Computational strategy

We base our approach on key observations from manual methods. From Wonnacott’s work, we know that focusing on non-problematic instances that are not connected to the updated tile is beneficial. From Li’s work [19], we observed that such instructions (*S*_1_) can be organized into an efficient schedule similar to matrix multiplication, which enables execution on ubiquitous parallel platforms.

Our experience with polyhedral compilers [9,16] allows us to use ISL tools for semi-automatic code generation and to automate parts of the process, including dependency management. Ultimately, our goal is to produce high-performance code that enables vectorization, as in classical approaches, so that the performance is comparable to the results achieved in Frid’s and Tchendji’s work [21,23] as well as polyhedral approaches [9,28].

#### Dependence pattern.

As we mentioned, using the ISL library, we can represent dependencies through relations, just like in Traco and Dapt. Additional transitive paths can be derived from these relations.

The positive transitive closure for a given relation *R*, *R*^+^, is defined as follows [25]:


$$\begin{array}{c} R^{+}=\{e\to e^{\prime }:\;e\to e^{\prime }\in R\;\vee \exists e^{\prime \prime }s.t.\;e\to e^{\prime \prime }\in R\;\wedge \;e^{\prime \prime }\to e^{\prime }\in R^{+}\}. \end{array}$$ (2)

It describes which vertices $e^{\prime }$ in a dependence graph (represented by relation *R*) are connected directly or transitively with vertex *e*.

Transitive reduction applied in Presburger arithmetic is an approach to simplify relationships (expressed as logical constraints) while preserving their fundamental properties, such as reachability. Kelly et al. described this approach called *Simple Redundant Synchronization Removal* in paper [32] as


$$\begin{array}{c} R^{-}:=R-(R^{+}\circ R). \end{array}$$ (3)

The composition of the relation *R*^+^ and *R*, or equivalently the application *R*^+^(*R*) [27], gives all paths between two nodes in the graph whose length is greater than or equal to 2. To compute the exact transitive reduction, we first need to compute the exact transitive closure of the union of all dependence graphs. It is worth noting that for Nussinov’s loop dependence relations, ISL [27] only approximates the transitive closure. However, we can use the iterative algorithm presented by Bielecki and Klimek in [33], which is capable of computing transitive relations without approximation for Nussinov’s loop dependences [9]. The tool was implemented within the TRACO compiler. We have placed the exact form of *R*^+^ for Nussinov’s loop nest dependencies in S2 Text.

Since some dependencies imply others, transitive reduction is applied to remove redundancies [24]. This leaves only a union of four dependence relations, reduced from seven, which simplifies the subsequent dependence analysis presented in the paper.


$$\begin{array}{c} \begin{array}{cc} R_{1}:= & \;S_{1}[i,j,k]\to S_{1}[i,j,1+k]\;:i\geq 0\wedge j<N\wedge 0\leq k\leq j-2-i\\ R_{2}:= & \;S_{2}[i,j]\to S_{1}[i,j^{\prime },-i+j]\;:i\geq 0\wedge j>i\wedge j<j^{\prime }<N\\ R_{3}:= & \;S_{1}[i,j,-1-i+j]\to S_{2}[i,j]\;:i\geq 0\wedge i<j<N\\ R_{4}:= & \;S_{2}[i,j]\to S_{1}[i-1,j,0]\;:i>0\wedge i<j<N \end{array} \end{array}$$ (4)

Among the presented relations, only one, *R*_1_, pertains to dependencies of instruction *S*_1_ to *S*_1_, and it represents the combined tile blocks in all three dimensions. The remaining dependencies represent the relation of instruction $S_{1}\to S_{2}$ within the same iteration *i*, *j*, and the dependency $S_{2}\to S_{1}$ along the row *i* and column *j*. This ensures that loop skewing naturally handles these dependencies by definition. Therefore, within the scope of our interest, only the *R*_1_ relation remains.

The relation *R*_1_ forms a chain of consecutive instructions (i,j,k),(i,j,k+1),(i,j,k+2),…, creating a sequence of *S*_1_ instructions, which is the only instruction triply nested in the Nussinov loop nest. Sacrificing the third dimension in favor of two-dimensional blocks enables vectorization and facilitates the separation of non-problematic blocks in the subsequent code.

#### Problematic and non-problematic tiles.

We can now imagine a square block of instructions computed simultaneously for fixed (*i*, *j*), with dimensions bb × bb, where the values are stored in individual elements of the block and later reused. The key question becomes: which instructions can be executed in any order, and which must not be reordered, as their execution is determined by dependencies within a specific block?

Each block is described by coordinates (*II*, *JJ*), and in order to compute it, we only use instructions that are not directly related to each other by dependence within that block.

Since our focus is on the *S*_1_ instruction, we must analyze its control flow. It performs two reads, from positions (*i*, *k*) and (*k* + 1, *j*), and writes to position (*i*, *j*). The write to (*i*, *j*) belongs to the currently computed tile; therefore, we are interested only in those instructions that do not directly access this tile.

**Fig 1:**
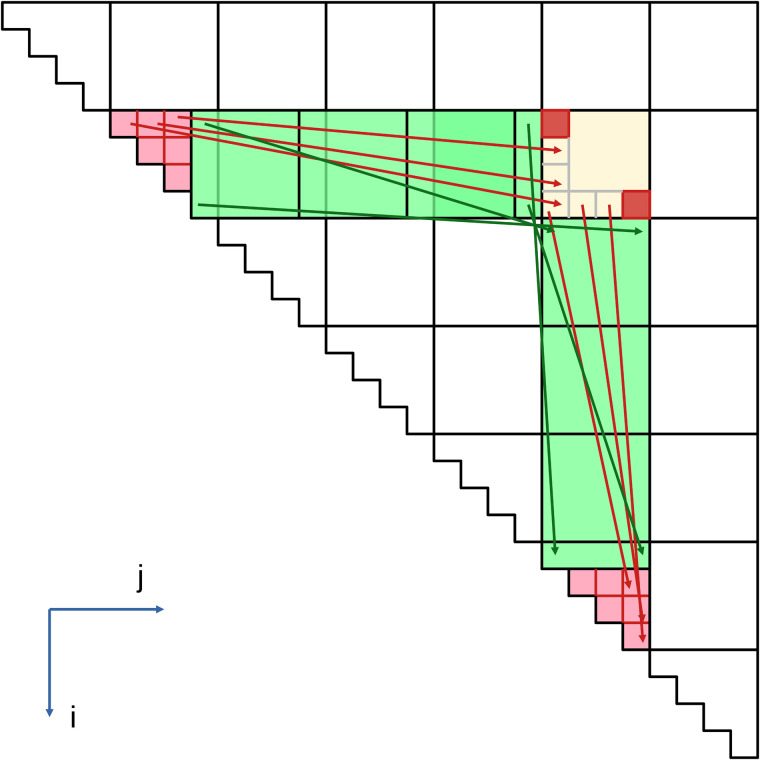
The problematic (red) and non-problematic (green) tiles, and the update tile (yellow) in Nussinov’s loop nest iteration space for the *S*_1_ statement.

Based on the analyses presented in the works [2,3,19], we illustrate in Fig 1 the distinction between *problematic* and *non-problematic tiles* in the iteration space of Nissunov’s loop nest. The *non-problematic tiles*, marked in green, are those for which the instructions *S*_1_ can be executed independently, i.e., in any order relative to other cells within the current block (highlighted in yellow as the *current* or *update tile*). Additionally, two example red cells are highlighted, for which the execution of *S*_1_ is required for the current step, as the read references i,k and k + 1,j come from the same *update tile*. These dependencies are associated with the instructions in the red triangular regions, which form the set of *problematic tiles* and constitute the potential sources of conflicts related to the execution order of instructions.

In our approach, *problematic tiles* refer to computations of pairs that interact with elements of the currently processed block. Naturally, this block must still be compared with the final result of the non-problematic tiles; however, their execution can be performed in an arbitrary order. It is worth noting that both problematic and non-problematic tiles contain intra-tile dependencies, which are indicated by red and green arrows, respectively in Fig 1. *Problematic tiles* additionally contain inner-tile dependencies, whereas *non-problematic tiles* rely only on values that have already been computed.

If we formulate the dependency *DEP* := (i,k)→(k+1,j), it becomes clear that either the source or the destination of this relation may belong to the currently computed tile. By analyzing Fig 1, we can observe that these cases correspond only to instructions within the triangular blocks. Therefore, all other blocks will not directly access the memory cells written by the tile (*II*, *JJ*).

Wonnacott, in his paper [18], demonstrated that for a given tile (*II*, *JJ*), a subset of instructions can be safely parallelized and executed in any order. He referred to these as “almost tileable” or simply *non-problematic* instructions. These correspond to the second group of instructions in our analysis—those that do not directly depend on or affect the memory cells of the current tile. Consequently, the remaining instructions, which involve dependencies that may include the current tile’s memory region, are referred to as *problematic*. First, let us define TILE(II,JJ)


$$\begin{array}{c} \begin{array}{cc} & TILE(II,JJ):=\{[ii,jj,i,j,k]:0\leq jj\leq \frac{N}{bb}\wedge 0\leq ii\leq \frac{N}{bb}\;\wedge \\ & 0\leq i<N\wedge 0\leq j<N\wedge 0\leq k<j-i\;\wedge \\ & ii\cdot bb\leq i<(ii+1)\cdot bb\wedge jj\cdot bb\leq j<(jj+1)\cdot bb\;\wedge ((ii=jj\wedge j>i)\vee jj>ii)\} \end{array} \end{array}$$ (5)

Wonnacott identified all “problematic” iterations — those that read from the tile being updated — by taking the intersection of each dependence relation with a relation whose source and sink iterations share the same values of all but the innermost loop [18].

Hence, we define the set of *problematic* instructions as follows:


$$\begin{array}{c} Z(II,JJ)=\text{DEP}(\text{TILE}(II,JJ))\cup {\text{DEP}}^{-1}(\text{TILE}(II,JJ)), \end{array}$$ (6)

that is, all instructions which either depend on the current tile or are depended on by it. It corresponds to relation *R*1 as a chain of *S*1 statements, but only those whose source or sink belongs to the current (updated) tile.

Consequently, the set of *non-problematic* instructions *Z*_*N*_ for the current tile is the domain of instruction *S*_1_ excluding *Z*, i.e.,


$$\begin{array}{c} \text{\$Z\_N\$(II,JJ)}=\text{Dom}(S_{1}(II,JJ))⧵Z(II,JJ). \end{array}$$ (7)

Within the set of *non-problematic* instructions, denoted *Z*_*N*_, we can further partition the instructions based on their tile coordinates (*II*, *JJ*, *KK*), where *II* < *KK* < *JJ*, following the domain of the triangular matrix used in the Nussinov algorithm.

Although the *DEP* relation represents the *Read after Read (RAR)* dependence, it is possible to determine a path based on the analysis of the R dependency from the Nussinov code. The cells *i*,*k* and *k* + 1,*j*, although they can be determined independently, are needed at the same time to determine *i*,*j*. If there are read-write dependencies i,k→i,j and k+1,j→i,j, we can determine the connecting path through the union of the transitive closure of the relation and its inverse, $R^{+}\cup (R^{+}{)}^{-1}$. In other words, the path can be found in the undirected graph of dependencies.

A special case of *problematic* instructions outside the set *Z* also includes the set of *S*_2_ instructions, which simply remain within the 2D blocking domain. Additionally, we must consider the triangular blocks along the first diagonal, since for these the set *Z*_*N*_ does not exist (they are defined by constraint (ii=jj∧j>i). It is worth noting that for the second diagonal as well, there will be no tiles satisfying the condition *ii* < *kk* < *jj*; only starting from the third diagonal does the set *Z*_*N*_ become non-empty.

At the end of this subsection, we should clearly define *problematic* and *non-problematic* tiles. A tile is considered *problematic* if it contains any *problematic* instructions from *Z*. Therefore, a *non-problematic* tile consists only of *non-problematic* instructions, in this case, only *S*_1_.

#### Result code.

Upon reanalyzing the condition *i* < *k* < *j*, or equivalently 0 ≤ *k* < *j* − *i*, we need to identify which *S*_1_ instructions belong to the set of *problematic* ones. It can be observed that these correspond to two specific regions:

- Instructions located within the triangular block of the current tile, i.e., those for which i<k<ii·bb−1,
- And the final part of the current tile where jj·bb−1<k<j.

These are the instructions whose dependencies either reach into or are affected by the memory region of the tile being currently computed.

A loop is considered parallel if it does not carry any dependence; that is, for all dependences (x→y), the corresponding dependence vector in the scheduled space has a zero component along that loop dimension. In practice, this information is extracted from the affine schedules generated by Pluto [28], although other polyhedral compilers can also be used.

In accordance with the work [16], we formulate the set of tiles (*ii*, *jj*) scanned along the diagonals, and generate the code, for example, using the Barvinok tool [34], which accepts interactive commands in ISL.


$$\begin{array}{c} \begin{array}{cc} & D:=\{[ii,jj,i,j,k]\to [jj-ii,jj,j-i,j,k]:constraints(TILE(II,JJ))\} \end{array} \end{array}$$ (8)

To generate the code, we use the codegen function from the Barvinok tool, which scans 2D block identifiers and the instructions inside each block. It is presented on Listing 2. The *c*_0_ loop sequentially iterates along diagonal lines, and within each block, computations are performed independently according to loop skewing.

**Listing 2. The result code for the Nussinov’s RNA folding computation**.

1

2 for (int c0 = 0; c0 <= (N – 1) / bb; c0 += 1) // serial loop

3 #pragma omp parallel for

4 for (int c1 = c0; c1 <= N / bb; c1 += 1) {

5   short C[bb][bb] = {0};

6   short _ii = c1 - c0;

7   short _jj = c1;

8

9   // non-problematic tiles

10  for (short kk = _ii + 1; kk < _jj; kk++)

11    for (int row = 0; row < bb; row++)

12 #pragma omp simd

13     for (int col = 0; col < bb; col++)

14      for (int k = 0; k < bb; k++)

15       s1(bb * _ii + row, bb * _jj + col, bb*kk + k – 1, &C[row][col]);

16

17   // square tiles with problematic s1 and s2

18   if (_jj >= _ii + 1) {// c0 >= 1

19    for (int c2 = bb * _ii + bb – 1; c2 >= bb * _ii; c2––)

20     for (int c3 = bb * _jj; c3 <= min(N – 1, bb * _jj + bb – 1); c3++) {

21      for (int c4 = c2; c4 < c2 + bb – 1; c4++)

22       s1(c2, c3, c4); // triangle boundary block

23

24      S[c2][c3] = max(C[c2 - bb * _ii][c3 - bb * _jj], S[c2][c3]);

25

26      for (int c4 = bb * _jj – 1; c4 < c3; c4++)

27       s1(c2, c3, c4); // current block

28

29      s2(c2, c3);

30     }

31   }

32   // triangular first diagonal blocks

33   else if (_jj == _ii) {// c0 = 0

34    for (int c2 = min(N – 2, bb * _jj + bb – 2); c2 >= bb * _jj; c2––)

35     for (int c3 = c2 + 1; c3 <= min(N – 1, bb * _jj + bb – 1); c3++) {

36      for (int c4 = c2; c4 < c3; c4++)

37       s1(c2, c3, c4);

38      s2(c2, c3);

39     }

40   }

41 }

**Listing 3. Statements code**.

1 inline void s1(int i, int j, int k) {

2  S[i][j] = max(S[i][k] + S[k + 1][j], S[i][j]);

3 }

4

5 inline void s1(int i, int j, int k, short *s) {

6  *s = max(S[i][k] + S[k + 1][j], *s);

7 }

8

9 inline void s2(int i, int j) {

10  S[i][j] = max(S[i][j], S[i + 1][j-1] + paired(seqq[i], seqq[j]));

11 }

The generated code is divided into two parts:

1. For *c*_0_ = 0, blocks are computed separately because the loop bounds form a triangular shape.
2. for *c*_0_ ≥ 1, the code generator computes square blocks sequentially. Originally, the code generator computed a loop for all *k* values. In this case, using the sets *Z*_*n*_, we extract the kk loops and instructions from this nest, compute blocks separately, and create a maximum value array *C* for each thread.

To enable tiling of the *k* loop, a thread-local array C is used to compute the max reduction. This design eliminates the intra-tile reduction dependence (*R*_1_), allowing the tiling transformation to be applied safely and efficiently.

Some parts of the code still require adjustments, but only on the *s1* statement. From the loop in line 19, we need to recompute the *c*_4_ loop (corresponding to *k*) up to the first triangular block, compare it with the value in the *C* array, and add the elements from the current block. This modification is applied manually, but knowledge of the sets *Z*_*n*_ and *Z* is helpful here. For example, for *TILE*(1,5) from Fig 1, Z=TILES{(1,1)→(1,5),(1,5)→(5,5)} while Zn={(1,2)→(2,5),(1,3)→(3,5),(1,4)→(4,5)}. So, in general, TILE(II,JJ) is combined with triangular tiles (II,II) and (JJ,JJ), called *problematic*. While *Z*_*n*_ for TILE(II,JJ) are pairs of tiles (II, KK) and (KK, JJ), where II < KK < JJ, which represents lines 10–15 in Listing 2.

To avoid a nonlinear expression, we introduce *bb* as a constant. Next, we exclude the set of *non-problematic* instructions from the set and proceed to compute the tiles (*ii*, *jj*, *kk*), previously saving the current results in a local array *C* in the block in cache (with each thread having its own copy). These values must be taken into account when processing the *problematic* instructions.

To simplify the final code, we provided inline functions representing *S*_1_ and *S*_2_ statements (Listing 3). The code defines three inlined helper functions used in the Nussinov RNA folding algorithm. Function s1(i, j, k) implements the dynamic programming recurrence relation: it updates S[i][j] with the maximum between its current value and the sum of S[i][k] and S[k + 1][j]. The overloaded version s1(i, j, k, short *s) performs the same computation but stores the result in a temporary variable pointed to by s, which is used to store values in the local *C* array. The function s2(i, j) handles the special case where positions i and j are paired: it updates S[i][j] based on whether the nucleotides at those positions can form a valid base pair, using the helper paired() function and the current values in the DP matrix.

This code presented on Listing 2 implements a parallel, tiled version of the Nussinov RNA folding algorithm using OpenMP and SIMD. It operates on a blocked dynamic programming matrix with tile size bb, processing diagonals in order to respect data dependencies. The outer loop (c0) runs serially over diagonals, while the inner loop (c1) is parallelized. Each thread computes a tile (ii, jj) of the matrix using a private local array C, ensuring no data races—each thread has its own C. *Non-problematic* tiles are processed with standard recurrence using s1(), leveraging SIMD for vectorization. Square and triangular tiles (on or near the diagonal) are handled separately due to irregular index ranges, combining s1() and s2() calls. The results are written back to the global matrix S, using max() to update optimal base pairings. To enable loop parallelism and vectorization, we used the #pragma omp parallel for and #pragma omp simd directives from OpenMP [31], respectively.

It is worth noting that the loops iterating over *row*, *column*, and *k* can be freely interchanged and vectorized, regardless of whether they are outer or innermost loops.

We observed that the proposed computational strategy can be applied to similar NPDP problems. In S1 Text, we present a brief analysis of a closely related OBST algorithm based on Knuth’s method.

## Experimental study

Experiments were conducted on modern machines, which are equipped with massively parallel units 2 × Intel Xeon Gold 6326, Intel CPU Max 9462, AMD Epyc 9654, and partially with 2 × Intel Platinum 8358.

The first machine includes two Xeon Gold 6326 processors, providing a total of 32 physical cores and 64 threads. Each processor operates at a base frequency of 2.9 GHz, with a boost clock of up to 3.5 GHz. The system is based on the Ice Lake architecture (x86_64), supporting both 32-bit and 64-bit modes, with a 46-bit physical address space.

The memory hierarchy includes 1.5 MB of L1 cache distributed across 32 instances, 40 MB of L2 cache split into 32 instances, and 48 MB of L3 cache divided into two instances. The system is equipped with 131 GB of RAM and runs on Ubuntu 22.04.4 LTS.

For compilation, the Intel ICC compiler 2025.3.0 was used to ensure optimized performance for the experiments with the flags -O3, -qopenmp, and -mavx512 for vectorization. In our comparisons, we did not include the implementations [2,18,20], as they are either unavailable or do not provide parallel versions.

Each experiment was repeated five times, and the reported results correspond to the arithmetic mean. The observed standard deviation is low, and for the largest problem sizes, the differences between runs remain below 5 seconds. Additionally, the relative variability, defined as (max−min)/mean×100%, is within a few percent for longer execution times. As expected, both the standard deviation and the relative variability increase for shorter execution times, which can be attributed to the limited resolution and inherent noise of timing measurements. The complete statistical analysis is provided in the Data Availability section.

Table 1 presents the results for polyhedral compilers, the *Transpose* technique [19], and the parallel and sparsified Four Russians (PS4R) with tiling (PTS4R) method [23]. The polyhedral compilers were provided with Listing 1 as input. The Pluto compiler does not tile the innermost loop *k*, as confirmed for releases 0.11.4, 0.12.8, and 0.13, which results in the poorest performance among the compared techniques (flags ––parallel ––tile were used). Traco applies transitive closure on the dependence graph and can tile all loop nests; however, the use of irregular tiles reduces the achieved speedup. The best-performing automatic compiler is Dapt, whose time-space tiled code achieves strong performance on the Nussinov algorithm. It is worth noting that the OneAPI compiler is unable to vectorize the code generated by Pluto, Traco, and Dapt, and it applies only loop unrolling.

**Table 1:** A performance comparison for Gold 6326 across varying input sizes (in thousands of nucleotides), with execution times expressed in seconds.

Size	Pluto	Traco	Dapt	Transpose	PS4R	PTS4R	Ours
1	0.05	0.21	0.22	0.04	0.06	0.05	0.02
2.5	0.43	1.55	1.54	0.34	0.35	0.36	0.06
5	4.34	7.35	7.19	4.92	2.29	2.20	0.13
7.5	16.31	20.30	19.32	19.45	7.58	7.52	0.36
10	129.15	42.67	44.52	47.13	16.82	15.37	1.13
15	439.29	140.86	153.67	153.26	56.86	46.74	3.96
20	–	352.14	368.61	335.59	140.14	113.84	7.57

The *Transpose* technique operates on the lower triangle of the matrix to enable row-wise access. However, the requirement for a double-sized array negatively impacts data locality, which limits its effectiveness—it only outperforms Pluto. The newly introduced PTS4R technique, which builds upon Traco’s loop tiling approach, delivers competitive performance. Nevertheless, untiled parallel sparsification PS4R yields better results overall.

In contrast, our implementation significantly outperforms all of the above methods. It is simple and concise, avoiding the complex loop-bound computations required by polyhedral tools. The code achieves high cache efficiency, and parallelization of the inner loops enables effective vectorization of regular instruction patterns. As a result, it delivers consistent performance improvements, especially at larger problem sizes. We empirically tested various block sizes. In most cases, a block size of 32 provided the best performance, both in our implementation and in those using polyhedral compilers. For the PTS4R method, however, the default block size of 128 proved to be more effective.

We repeated the experiments with a second machine equipped with an Intel Xeon CPU Max 9462 (the Sapphire Rapids HBM architecture), featuring 64 hardware threads, up to 3.5 GHz turbo, a 150 MB L3 cache, a 128 MB L2 cache, a 3 MB L1d cache and a 2 MB L1i cache, as well as 128 GB of on‑package HBM2e and 631 GB of DRAM. The code was compiled with the Intel oneAPI C++ Compiler 2025.0.4 using the flags -O3, -qopt-report = max, -qopt-report-phase = vec, -march = native, -mavx512, and -qopenmp. The resulting .optrpt report indicates that vectorization was successfully enabled for the loop responsible for scanning columns in the non-problematic instance stream, with a vector length of 32 (see S3 Text).

The second machine is equipped with larger local memory, which is reflected in the results. The Dapt compiler starts to outperform both Pluto with 2D tiling and the *Transpose* technique only beyond input sizes of 10,000 nucleotides. However, techniques based on sparsified Four Russians continue to dominate, with the untiled parallel version showing a clear advantage (Table 2). On the other hand, our implementation demonstrates more stable performance across all input sizes and significantly outperforms the related methods mentioned above.

**Table 2:** A performance comparison for CPU Max 9462 across varying input sizes (in thousands of nucleotides), with execution times expressed in seconds.

Size	Pluto	Traco	Dapt	Transpose	PS4R	PTS4R	Ours
1	0.10	0.43	0.25	0.07	0.11	0.11	0.02
2.5	0.36	2.20	2.81	0.29	0.48	0.62	0.08
5	2.47	8.18	8.53	2.57	2.05	2.09	0.32
7.5	9.19	19.09	21.00	9.18	5.91	9.34	0.75
10	61.01	34.31	37.73	29.14	13.60	18.64	1.06
15	193.41	100.45	106.70	88.08	46.55	63.69	3.25
20	–	216.38	234.35	250.97	98.91	140.13	7.04

We extended our research by including a machine, this time from AMD — the AMD Epyc 9654 96-Core Processor, featuring 192 threads, 384MB of shared L3 cache, 1MB of L2 cache per core, and 64KB of L1 cache per core. The processor boosts up to 3.7 GHz, is equipped with 1.5 TiB of RAM, and runs AlmaLinux 8.7. The codes were compiled in the same manner as on the CPU Max machine, using the OneAPI compiler with version 2024.0.2.

**Fig 2:**
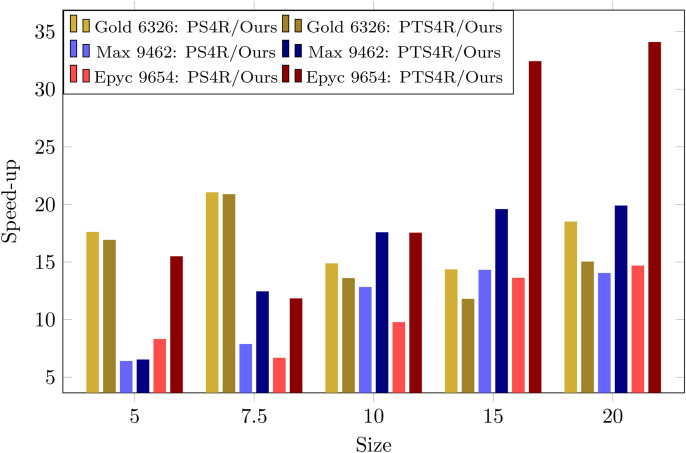
Speed-ups of our approach with respect to PS4R and PTS4R across different CPUs and input sizes (in thousands of nucleotides).

Once again, our execution times turned out to be the shortest (Table 3). On AMD architectures, 2D tiling of Pluto and Transpose outperforms 3D tiling because it makes better use of the cache hierarchy and reduces synchronization overhead. 2D tiles create smaller, more cache-friendly blocks of data, while 3D irregular tiles can exceed cache capacity, causing more memory accesses. Additionally, 2D loops tend to generate more regular memory access patterns, which improves SIMD utilization and prefetching, resulting in higher overall performance. Pluto stood out as the fastest among the tested polyhedral compilers. The *Transpose* implementation by Li’s team [19] performed well, despite using twice the memory, and lagged behind the parallel PS4R method for RNA strands over 15,000 nucleotides. Fig 2 shows that our approach outperforms the best related methods, PS4R and PTS4R, achieving consistent speed-ups ranging from 5× to over 30× across input sizes on various CPU architectures.

**Table 3:** A performance comparison for Epyc 9654 across varying input sizes (in thousands of nucleotides), with execution times expressed in seconds.

Size	Pluto	Traco	Dapt	Transpose	PS4R	PTS4R	Ours
1	0.03	0.15	0.21	0.04	0.06	0.08	0.02
2.5	0.26	0.98	1.43	0.17	0.27	0.38	0.04
5	1.75	4.34	6.16	0.82	1.33	2.48	0.16
7.5	5.46	10.96	13.99	2.45	4.21	7.46	0.63
10	26.87	50.95	42.29	6.04	8.02	14.39	0.82
15	79.02	137.58	98.25	32.68	26.03	61.97	1.91
20	189.98	295.54	222.56	91.88	58.17	135.06	3.96

Therefore, it can be concluded that the presented implementation is the fastest among CPU-based available solutions for Nussinov’s code, which also suggests potential for strong performance on other OpenMP-based platforms such as ARM. It falls short when compared to the corresponding blocked CUDA version, which was analyzed in [16]. The GPU implementation was optimized to perform loop skewing by launching kernels on diagonal blocks, enabling 2D parallel execution of loops iterating over the rows and columns of the matrices, and storing the output matrix C in shared memory.

In Table 4, we present a comparison of the proposed CPU implementation on Xeon CPU Max 9462 (64 threads), 2 × Platinum 8358 (2 × 64 = 128 threads), and AMD Epyc 9654 (192 threads) with CUDA implementations from the paper [16] on selected NVIDIA cards A100, V100, and RTX 4060. It is clear that, despite the inherently less parallel architecture of the CPU, the use of vectorization and data locality enables the CPU-based code to achieve performance results that are relatively close to those of the more parallelized GPU implementation, especially with the AMD Epyc machine.

**Table 4:** Execution times (in seconds) of the CUDA and OpenMP implementations across NVIDIA GPU and Intel/AMD CPU platforms for varying input sizes (in thousands of nucleotides). The best results for each architecture are underlined.

Size	Tesla	Tesla	GeForce	Xeon CPU	2 x Xeon	Epyc
	A100	V100	RTX 4060	Max 9462	Platinum 8358	9654
5	0.33	0.19	0.18	0.32	0.57	0.16
7,5	0.48	0.44	0.50	0.75	0.74	0.63
10	0.79	0.81	1.02	1.06	1.19	0.82
15	1.61	2.10	3.00	3.37	2.91	1.91
20	3.29	4.90	6.56	7.78	6.48	3.96
30	8.62	14.33	20.63	23.63	20.55	10.72

In Table 5, we compare the parallel tiled sparsified 4R (PTS4R) OpenCL implementation executed on NVIDIA A100 and RTX 4060 GPUs with our proposed OpenMP implementation on Xeon Gold, CPU Max, and Epyc 9654 processors. The non-tiled PS4R version was not implemented by Tchendji et al. on GPU [14]. The execution time results indicate that the proposed approach consistently outperforms the related method.

**Table 5:** Execution times (in seconds) for varying input sizes (in thousands of nucleotides), comparing the OpenCL-based PTS4R implementation on GPUs with the OpenMP-based proposed implementation on CPUs.

Size	A100	RTX 4060	Gold 6326	CPU Max 9462	Epyc 9654
	PTS4R GPU		Ours		
1	0.38	0.20	0.02	0.02	0.02
2.5	0.63	0.50	0.06	0.08	0.04
5	2.13	2.67	0.13	0.32	0.16
7.5	7.31	7.82	0.36	0.75	0.63
10	17.30	23.00	1.13	1.06	0.82
15	50.23	65.82	3.96	3.25	1.91
20	112.31	149.13	7.57	7.04	3.96

## Conclusion

In this paper, we presented an optimized implementation of the Nussinov algorithm, characterized by high efficiency, locality, and vectorization. The solution is compact, concise, and tiled across all dimensions, providing a robust and scalable approach to RNA secondary structure prediction. We compared the execution times of our implementation on massively parallel CPU machines from Intel and AMD against both manually optimized solutions and those obtained from automatic compilers based on the polyhedral model. Our solution achieves speedups of at least 5× and up to over 30 × compared to related CPU-dedicated implementations of the Four-Russians method (PS4R and PTS4R) in OpenCL for the Nussinov algorithm that do not utilize Advanced Vector Extensions (AVX). Notably, even on CPU architectures, our implementation achieves speedups of 15× or more compared to the PTS4R GPU implementation.

The proposed approach is relevant to the class of NP-hard problems, and similar dependency structures and memory access patterns can be found in other algorithms within computer science and bioinformatics. Additionally, we demonstrated the dynamic nature of the algorithm, which presents ongoing challenges for optimization. Our work contributes to the field of semi-automatic transformations, as we leveraged ISL-based computations alongside insights from manual solutions available in the literature. In summary, dynamic programming tasks in bioinformatics executed on CPUs must be heavily vectorized to achieve results within a reasonable time.

Future research will aim to extend these methods to more advanced algorithms from the NPDP Benchmark Suite [10] and be dedicated to RNA folding, to further enhance computational efficiency in the bioinformatics domain. Additionally, we plan to investigate energy efficiency and quantify potential energy savings achieved by the proposed approach.

## Supporting information

S1 Table NPDP benchmarks similar to Nussinov’s code. (PDF)

S1 Text Short analysis of the NPDP Knuth algorithm (OBST). (PDF)

S2 Text Exact transitive closure of the dependence graph for the Nussinov loop nest in ISL format. (PDF)

S3 Text Fragment of the Intel oneAPI C+ Compiler report showing loop vectorization in a non-problematic domain. (PDF)
