# Supplementary material for: Cache-efficient and vectorized parallel dynamic programming for RNA folding
Source: PLoS One. 2026 May 20;21(5):e0349146. doi: 10.1371/journal.pone.0349146 (PMC13189310; doi:10.1371/journal.pone.0349146)
Supplement: S1 Table — (PDF) [file pone.0349146.s001.pdf]

S1 Table. NPDP benchmarks similar to Nussinov's code on which the proposed approach can be applied

**Matrix Chain Multiplication**<br>$m[i,j] = \min_{i \leq k < j}(m[i,k] + m[k+1,j] + p[i-1]p[k]p[j])$
**Optimal Binary Search Tree (OBST)**<br>$c[i,j] = \min_{i \leq k \leq j}(c[i,k-1] + c[k+1,j] + \sum_{r=i}^{j} w_r)$
**Polygon Triangulation**<br>$C(i,j) = \min_{i<k<j}(C(i,k) + C(k,j) + w(i,j,k))$
**Nussinov RNA Folding**<br>$S(i,j) = \max(S(i+1,j-1) + \sigma(i,j), \max_{i<k<j}(S(i,k) + S(k+1,j)))$
**McCaskill Partition Function**<br>$Q_{i,j} = Q_{i,j-1} + \sum_{i \leq k < j-l} Q_{i,k-1} Q_{k+1,j-1} \exp(-E_{bp}/RT) pair(k,j)$
