# Supplementary material for: Cache-efficient and vectorized parallel dynamic programming for RNA folding
Source: PLoS One. 2026 May 20;21(5):e0349146. doi: 10.1371/journal.pone.0349146 (PMC13189310; doi:10.1371/journal.pone.0349146)
Supplement: S1 Text — (PDF) [file pone.0349146.s002.pdf]

S1 Text. Short analysis of the NPDP Knuth algorithm (OBST)

The loop from [1] is structurally similar to the Nussinov loop nest, but simpler:

```
#pragma scop
for(i=n-1; i>=1; i--)
  for(j=i+1; j<=n; j++)
    for(k=1; k<j-i; k++)
      ck[i][j] = MIN(ck[i][j], w[i][j]+ck[i][k+i]+ck[k+i][j]);
#pragma endscop
```

The loop nest contains a single statement (S_1), and the loop bounds for indices i and j differ only slightly. The computation involves a triangular iteration space, loop skewing, and a MIN reduction.

After transitive reduction, only one chain dependence relation remains, with vector $(0, 0, +)$. The input dependence is:

$$R := S_1[i, j, k] \to S_1[i, j, k'] : i > 0 \wedge j \leq n \wedge k > 0 \wedge k < k' < -i + j.$$

After reduction:

$$R := S_1[i, j, k] \to S_1[i, j, k + 1] : i > 0 \wedge j \leq n \wedge 0 < k \leq -2 - i + j.$$

The algorithm operates over a triangular domain where each cell $ck[i][j]$ depends on $ck[i][k + i]$ and $ck[k + i][j]$, inducing dependencies with vectors $(0, +, *)$ and $(+, 0, *)$.

When computations are performed along diagonals (i.e., for constant $j - i$), each diagonal depends only on previously computed diagonals. Therefore, there are no intra-diagonal dependencies, and parallelization along diagonals is valid. The original dependencies are preserved, and only the $(0, 0, +)$ relation needs to be considered.

Thus, the associative and commutative MIN reduction is valid for array C.

The key difference compared to the Nussinov algorithm lies in the RAR dependence:

$$DEP = [i, k + i] \to [k + i, j],$$

while for Nussinov it is:

$$DEP = [i, k + i] \to [k + i + 1, j].$$

This implies that the access shifts by one element, which affects non-problematic instances and requires a minor adjustment of the inline function. The overall code generation procedure remains analogous to the Nussinov case.

Reference

References

[1] Bielecki W, Blaszynski P, Poliwoda M. 3D parallel tiled code implementing a modified Knuth's optimal binary search tree algorithm. *Journal of Computational Science*. 2021;48:101246. doi:10.1016/j.jocs.2020.101246.
