# Supplementary material for: Cache-efficient and vectorized parallel dynamic programming for RNA folding
Source: PLoS One. 2026 May 20;21(5):e0349146. doi: 10.1371/journal.pone.0349146 (PMC13189310; doi:10.1371/journal.pone.0349146)
Supplement: S2 Text — (PDF) [file pone.0349146.s003.pdf]

S2 Text. Exact transitive closure of the dependence graph for the Nussinov loop nest in ISL format

```
[N] -> {
[i, j, i2, s1] -> [i', j', o2, s1] : 0 <= i2 < -i + j and i' >= 0 and j < j' < N and
  ((0 <= o2 <= -2 + i - i') or (i' < i and i - i' <= o2 < -i' + j'));
[i, j, i2, s1] -> [i', j' = j, 0, s2] : j < N and 0 <= i2 < -i + j and 0 <= i' <= i;
[i, j, i2, s1] -> [i', j', 0, s2] : 0 <= i2 < -i + j and 0 <= i' <= i and j' < N and
  ((i' <= -2 + i and j' > j) or (i' >= -1 + i and j' >= i + j - i'));
[i, j, i2 = 0, s2] -> [i', j' = j, o2, s1] : j < N and 0 <= i' < i and i-i' <= o2 < j - i';
[i, j, i2 = 0, s2] -> [i', j', o2, s1] : j > i and i' >= 0 and j' < N and     ((j' >= j and 0 <= o2
     < i - i') or (i' <= -2 + i and j' > j and i - i' <= o2 < -i' + j')     or (-1 + i <= i' <= i
    and j - i' <= o2 < -i' + j'));
[i, j, i2, s1] -> [i' = i, j' = j, o2, s1] : i >= 0 and j < N and i2 >= 0 and i2 < o2 < -i + j;
[i, j, i2 = 0, s2] -> [i' = -1 + i, j', o2, s1] : i>0 and j < j' < N and 0 < o2 <= -i +j;
[i, j, i2, s1] -> [i' = i, j', o2, s1] : i>=0 and 0 <= i2 < -i + j and j' < N
 and -i + j <= o2 < -i + j';
[i, j, i2, s1] -> [i', j' = j, o2, s1] : j < N and 0 <= i2 < -i+j and
  0 <= i' < i and 0 <= o2 < j - i';
[i, j, i2, s1] -> [i', j', -1+i-i', s1] : 0 <= i2 < -i+j and 0 <= i' < i and j<= j' < N;
[i, j, i2 = 0, s2] -> [i', j', 0, s2] : j > i and 0 <= i' <= i and j' >= j
  and -i + j + i' < j' < N }
```
