# Supplementary material for: Cache-efficient and vectorized parallel dynamic programming for RNA folding
Source: PLoS One. 2026 May 20;21(5):e0349146. doi: 10.1371/journal.pone.0349146 (PMC13189310; doi:10.1371/journal.pone.0349146)
Supplement: S3 Text — (PDF) [file pone.0349146.s004.pdf]

S3 Text. Fragment of the Intel oneAPI C++ Compiler report showing loop vectorization in a non-problematic domain

```
LOOP BEGIN at nuss_best.cpp (114, 1)
remark #15301: SIMD LOOP WAS VECTORIZED
remark #15305: vectorization support: vector length 32
remark #15389: vectorization support: unmasked unaligned unit stride load: [ /home/user/nuss_best.
    cpp (117, 81) ]
remark #15389: vectorization support: unmasked unaligned unit stride store: [ /home/user/nuss_best.
    cpp (37, 0) ]
remark #15475: --- begin vector loop cost summary ---
remark #15476: scalar cost: 22.000000
remark #15477: vector cost: 4.031250
remark #15478: estimated potential speedup: 5.421875
remark #15309: vectorization support: normalized vectorization overhead 0.000000
remark #15570: using scalar loop trip count: 32
remark #15488: --- end vector loop cost summary ---
remark #15447: --- begin vector loop memory reference summary ---
remark #15450: unmasked unaligned unit stride loads: 1
remark #15451: unmasked unaligned unit stride stores: 1
remark #15474: --- end vector loop memory reference summary ---
```
